# A Convolutional Neural Network for Compound Micro-Expression Recognition

**DOI:** 10.3390/s19245553

**Published:** 2019-12-16

**Authors:** Yue Zhao, Jiancheng Xu

**Affiliations:** School of Electronics and Information, Northwestern Polytechnical University, Xi’an 710129, China; xujchg@nwpu.edu.cn

**Keywords:** compound micro-expressions, EVM, FACS, 3D-FFT, TV-L1 optical flow, CNN

## Abstract

Human beings are particularly inclined to express real emotions through micro-expressions with subtle amplitude and short duration. Though people regularly recognize many distinct emotions, for the most part, research studies have been limited to six basic categories: happiness, surprise, sadness, anger, fear, and disgust. Like normal expressions (i.e., macro-expressions), most current research into micro-expression recognition focuses on these six basic emotions. This paper describes an important group of micro-expressions, which we call compound emotion categories. Compound micro-expressions are constructed by combining two basic micro-expressions but reflect more complex mental states and more abundant human facial emotions. In this study, we firstly synthesized a Compound Micro-expression Database (CMED) based on existing spontaneous micro-expression datasets. These subtle feature of micro-expression makes it difficult to observe its motion track and characteristics. Consequently, there are many challenges and limitations to synthetic compound micro-expression images. The proposed method firstly implemented Eulerian Video Magnification (EVM) method to enhance facial motion features of basic micro-expressions for generating compound images. The consistent and differential facial muscle articulations (typically referred to as action units) associated with each emotion category have been labeled to become the foundation of generating compound micro-expression. Secondly, we extracted the apex frames of CMED by 3D Fast Fourier Transform (3D-FFT). Moreover, the proposed method calculated the optical flow information between the onset frame and apex frame to produce an optical flow feature map. Finally, we designed a shallow network to extract high-level features of these optical flow maps. In this study, we synthesized four existing databases of spontaneous micro-expressions (CASME I, CASME II, CAS(ME)^2^, SAMM) to generate the CMED and test the validity of our network. Therefore, the deep network framework designed in this study can well recognize the emotional information of basic micro-expressions and compound micro-expressions.

## 1. Introduction

A micro-expression (ME) is an involuntary facial movement lasting less than one fifteenth of a second which can betray “hidden” or secretive emotions that the expresser attempts to conceal [1]. ME analysis has become an effective means to detect deception, and has many potential applications in the security field. Unlike macro-expressions, physiological studies have shown that MEs occur over a remarkably brief duration with only minor muscle changes, which makes them very challenging to detect and recognize [2]. To date, most facial micro-expressions research has focused on the study of six emotions typically seen in most cultures: happiness, surprise, anger, sadness, fear, and disgust [3,4,5,6].

Psychologists agree that understanding different types of facial expressions is essential for developing human emotional cognition and generating human-computer interaction models [7]. Though the majority of individuals can indeed recognize many different kinds of emotions, researchers have been limited to the six basic emotional categories [8,9]. Individuals create an abundance of facial expressions throughout their daily lives as they express their inner states and psychological needs [10]. For example, people regularly produce a happily surprised expression and observers do not have any problem distinguishing it from a facial expression of angrily surprised

**Figure 1:**
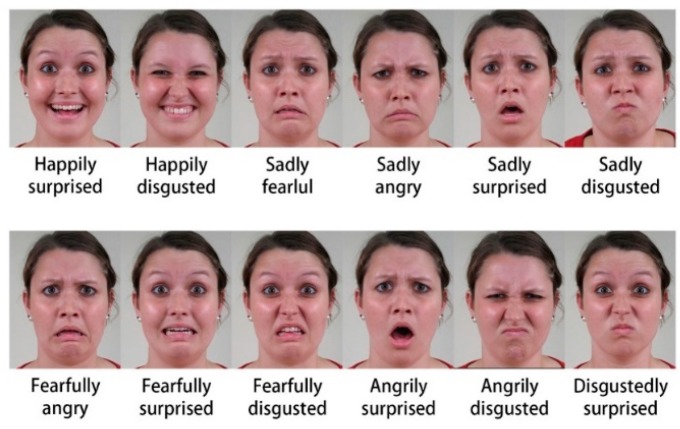
This Compound Facial Expressions of Emotion (CFEE).

Martinez et al. [11] created a group of compound expressions in an effort to expand human emotions based on computer vision. For example, as shown in Figure 1, “happily surprised” and “angrily surprised” are two typical complex emotions. They share the same emotion “surprise” but there are sufficiently different of muscle activations between each other. Martinez and his team defined 12 different compound emotions most typically expressed by humans, and generated a database called Compound Facial Expressions of Emotion (CFEE) [11]. Here, compound means that the emotion category is constructed as a combination of two basic emotion categories. Compound expressions are as important to human communication as basic expressions. Individuals through, and are acutely affected by, multiple emotions in their daily routine. Therefore, the usage frequency of compound expressions may be much higher than the research community has previously believed. For example, if somebody receives an unexpected gift, they will create a mixture of surprise and happiness which corresponds to the complex facial expression “happily surprised”.

In general, most ME recognition focuses on six basic emotions common in popular culture [1]. Unlike ordinary expressions (i.e., macro expressions), the psychological state reflected by MEs is often the closest to the real emotion of human beings. Consequently, MEs should not only be associated with single basic emotional categories, but also expand the diversity in emotional classes. Therefore, we need to build a ME database based on complex emotions. That is to say, “sadly angry” is a discrete category with unique facial representation compared to “angry” or “sadness”. Compound MEs are generally composed of two basic MEs. Furthermore, each externalized emotion has a corresponding unique facial expression, which is composed of the same muscle movements in individuals of different cultures and backgrounds [11]. In this paper, we generated a compound ME database (CMED) on the basis of five basic ME databases [12,13,14,15,16] which are cross-culturally applicable. As a result, the CMED database will aid researchers to train and evaluate their algorithm.

The extant research on ME recognition has centered on feature extraction [17,18]. Existing feature extraction methods can be roughly divided into two categories: hand-crafted feature-based methods [3,4,17] and deep-learning-based methods [19,20,21]. The former algorithms have been widely used in ME recognition systems, especially the LBP-family feature extraction method. The LBP family includes Local Binary Pattern on Three-Orthogonal Planes (LBP-TOP) [3], Local Binary Pattern with Six Intersection Points (LBP-SIP) [17], Spatiotemporal Local Binary Pattern with Integral Projection (STLBP-IP) [22], and Spatiotemporal Completed Local Quantization Patterns (STCLQP) [23]. Researchers have also applied optical flow methods to the study of MEs [5,6]. Optical flow involves the use of a strain mode to process continuous and changing videos, ultimately resulting in segmentation and extraction of ME sequences [24]. This method is robust to non-uniform illumination and large amounts of movement, but the optical flow estimation is strongly dependent on precise quantities of facial surface deformation [25]. Most of manual feature extraction methods, such as LBP-family and optical flow, are designed according to the characteristics of ME images. So they have high pertinence, weak generalization ability, and cannot well reflect the very small muscle changes associated with MEs.

In recent years, the emergence of deep learning methods has brought new vitality to the field of computer vision. Deep learning has been applied in research on face recognition [26], emotion recognition [27], and semantic segmentation [28] with remarkable results. There are two main methods for applying the Convolutional Neural Network (CNN) to macro-expression recognition. One involves constructing a complete recognition framework with the CNN [29], the other uses the CNN only for feature extraction [30]. Deep learning may be a very valuable tool to resolve the problem of ME recognition under traditional methodologies. However, most deep learning algorithms require a large number of databases for training in order to obtain representative features at the risk of over-fitting. As of now, there are not enough ME database samples for deep learning training. To this effect, most CNN-based ME recognition methods are based on the second method mentioned above. In most cases, the CNN is used to select ME deep features as proposed by Patel et al. [31], who first applied a CNN model to “transfer” information from object to facial expression, then secure useful depth features for recognition. Kim et al. [32] also proposed a multi-objective based spatial-temporal feature representation method for ME which functions well on the expression database MMI and the ME database CASME II.

The automatic ME recognition system is operated in roughly three steps: (1) image preprocessing, which serves to eliminate useless information from ME images and simplify the data; (2) feature extraction, which serves to extract useful information for machine learning from the original image; and (3) facial expression classification to recognize emotional categories based on extracted features. Each step plays an important role in the ME recognition process. Many researchers have established three-step ME recognition systems, the three steps may differ among them according to different requirements. Automatic ME recognition systems based on CNNs are still being only preliminarily explored.

**Figure 2:**
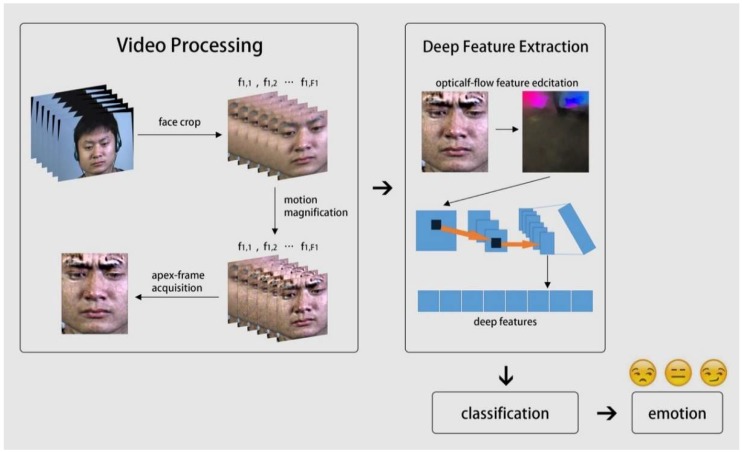
The framework of the proposed method.

This paper first proposes and synthesis the compound MEs based on computer vision methods and psychological studies. Moreover, a novel and robust feature extraction method for basic and compound MEs is presented. This algorithm not only effectively displays very slight muscle contraction motions, but also extracts the high-level features of MEs. An overview of the recognition system is provided in Figure 2. The main contributions of this study can be summarized as follows:

(1) The EVM algorithm is applied on the basic ME sequence to make the muscle movements more visible. In this way, we clearly understand the elicitation mechanism of MEs and use the corresponding action units to synthesize the compound MEs.
(2) The apex frames which include main information of ME sequences are extracted by 3D-FFT algorithm. The optical flow feature map between this frame and the onset frame was used to express the motion characteristics.
(3) A shallow CNN is used to extract high-level features from the flow feature map and classify the emotion classes.
(4) The proposed method was compared with several state-of-the-art methods to test the consistency and validity of the algorithm.

The remainder of the paper is organized as follows: Section 2 discusses the related works which include the generation of compound ME database, EVM technology, and the extraction of optical flow features and deep features. Section 3 discusses our experimental settings and results. Section 4 contains a brief summary and conclusions.

## 2. Proposed Method

### 2.1. Compound Emotion and MEs

Psychologists have posed countless questions about facial expressions as they relate to human emotions: How many kinds of expressions can people make? Does every facial expression represent a sign of emotion directly? Can a person lie using their expressions similarly to lying verbally? In approaching these questions, researchers have given us a deeper understanding of human emotional states [10,11].

Darwin first proposed six categories of emotions that are easily recognized by individuals regardless of their cultural background. The six emotion categories listed in the preceding paragraph are sometimes called “basic”. However, emotion categories should not be associated with a single feeling. Ekman found that there are more than 10,000 human facial expressions recognized by individuals to transmit information and psychological situation [10]. Experiencing a happily surprising event results in very different expression than those experienced when we are happy but not surprised. This compound feeling of happy and surprise is what we call a compound emotion. Furthermore, the externals of these emotional states are what we call compound expressions.

**Figure 3:**
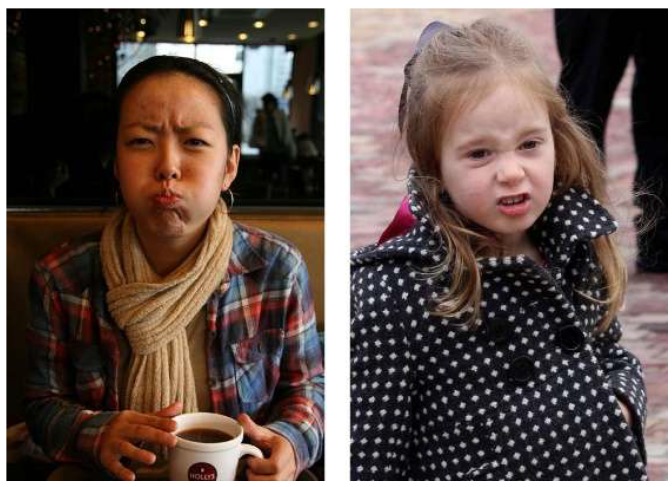
Compound facial expressions in real environments (**left**: “disgustedly surprised”, **right**: “fearfully surprised”).

Key to understanding compound expressions is to note that these categories are as important as the basic ones. As shown in Figure 3, “fearfully surprised” is a discrete category with a unique facial expression, and is different from “disgustedly surprised”, even though “surprise” is a common denominator in these emotions.

Ekman and Friesen defined a coding system that makes for a clear, compact representation of the muscle activation of a facial expression [33]. Their Facial Action Coding System (FACS) is given by a set of action units (AUs) [34]. Each AU codes the fundamental actions of individual or groups of muscles typically seen while producing facial expressions of emotion [35]. For example, AU1 represents the movement that pulls the inner portion of the eyebrows upwards. This kind of movement is involuntary and usually expresses negative emotions like surprise, fear, and sadness. The psychological basis of FACS provides a theoretical method for our experiments to synthesize compound ME images.

Martinez et al. [10] found 19 different facial movements related to emotions (one neutral expression + six basic expressions + 12 compound expressions) in the process of studying complex emotions, then generated the CEFF accordingly. Each compound facial expression in the CEFF is composed of two basic expressions. The difference lies in which emotional state is expressed more intensely. For example, in the compound emotion of “happily adjusted”, the latter expression is emphasized. Martinez and his team also analyzed the facial expressions of compound emotions made by people of different cultures. And then manually identified the AUs that were employed to express each emotion category. A muscle movement analysis of these expressions is shown in Table 1 [10].

All emotional categories have a unique facial expression. Table 1 shows that the AUs used to express complex emotions are consistent with those used to form basic emotions, and there is a certain affiliation between them. For example, the AUs that produce “happily disgusted” and “happily surprised” are all made up of the same AUs that create the emotion of “happy”. This phenomenon illustrates that the combination of basic emotions produced complex expression categories.

As shown in Table 1, we use “%” to indicate the proportion of participants using a given AU. There is a subordinate relationship between a compound expression and basic expression in terms of the AU. Sometimes when producing a compound expression, there is a conflict between the two basic expressions that synthesize the compound emotion. For example, a tight lip (AU 26) can be used to express disgust while lip angle elevation (AU 12) expresses happiness, but when expressing “happily disgusted”, AU 12 is used as more typical AUs express disgust while AU 26 automatically disappears. In this paper, we applied this theory to ME recognition, tailors and synthesizes our own compound ME database. Table 2 shows the muscular action units that people call when they produce MEs.

The approaches of generating and analyzing compound MEs were based on psychological studies. Based on the AUs theory shown in Table 2, this paper innovatively synthesizes compound ME images. It can be seen from Table 1 and Table 2, MEs correspond to the same AUs as macro-expressions. However, MEs are subtle and highly localized expressions of muscle movements. Therefore, we can use the AUs shown in Table 1 to synthesizes compound ME images.

**Figure 4:**
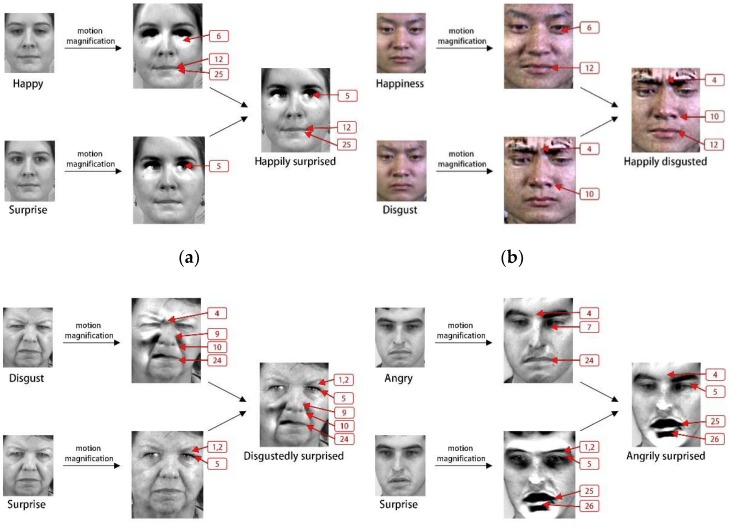

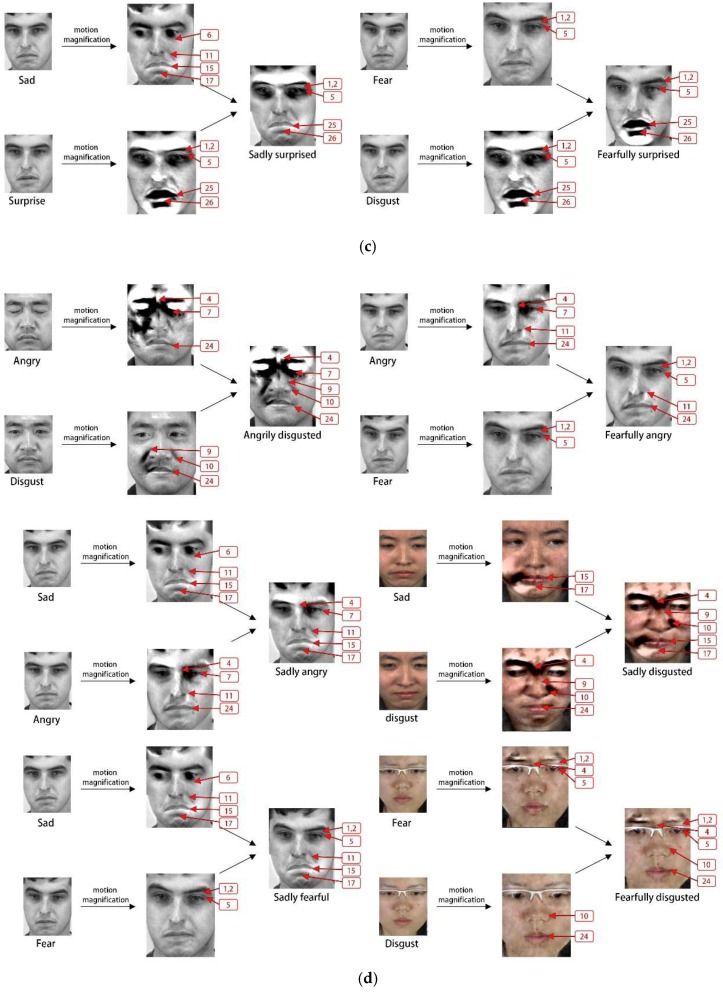
The generation process of CMED: (**a**) Description of Positively Surprised; (**b**) Description of Positively Negative; (**c**) Description of Negatively Surprised; (**d**) Description of Negatively Negative.

The subtle movements of basic ME sequences make difficult to identify and mark the AUs in the images. Based on this, the proposed algorithm introduces EVM method (details in the next section) to enlarge the facial structure of these ME sequences. As shown in Figure 4, we first analyzed the muscle contractions in these basic ME images and labeled the AUs accordingly. Moreover, we synthesized the compound MEs according to the theory of FACS and the distribution of AUs in Table 1 and Table 2. Finally, based on the compound expression theory [11] and Ekman’s knowledge of the FACS [33], we synthesized the first compound ME database (CMED) according the four existing databases of spontaneous MEs (CASME Ⅰ, CASME Ⅱ, CAS(ME)^2^, SAMM) in this study.

**Figure 5:**
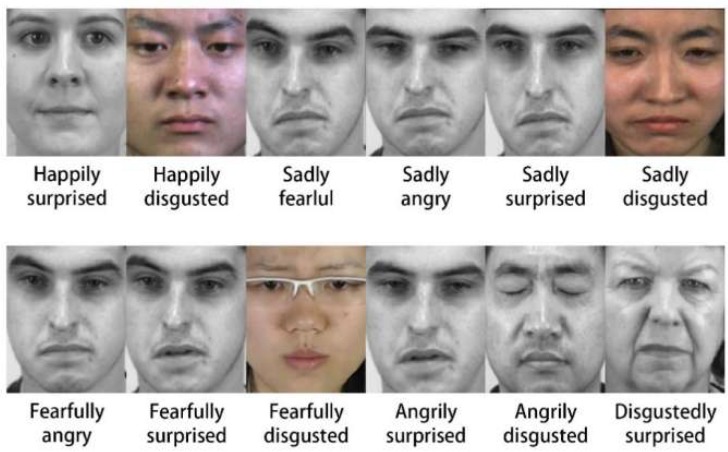
The compound micro-expression database.

As shown in Figure 4, we classify 12 compound MEs into four categories: Positively Surprised (PS), Positively Negative (PN), Negatively Surprised (NS) and Negatively Negative (NN). The generation information of compound MEs is also illustrated in detail. In Figure 4, we also see that the AU distribution of compound MEs is a combination of two basic emotions. For instance, sadly fearful is consistent with the state of sadness and fear. There are some mutual exclusions of AUs in this figure. For example, AU6, which is used to express sad eye, does not appear in the corresponding compound ME. The facial expression of sadly fearful emphasizes fear eye and sad mouth, so we automatically ignore the influence of sad eye in this generation process. Each basic emotion can only affect the generation of compound MEs regionally. Figure 4 depicts the compound ME images operated by EVM method. We also provide a group of original compound ME images shown in Figure 5. The CMED was established to extend the dimensions of ME recognition and provide data-support for researchers to carry out more complex research.

### 2.2. Image Preprocessing

As discussed in this section, we first preprocessed the compound ME to make it more effective in regards to feature extraction. Then, this paper estimated the optical flow motion of the onset frame and apex frame and generated the optical flow feature map. Finally, we used a shallow CNN network to extract the high-level features of these optical flow maps for ME recognition.

#### 2.2.1. Motion Magnification

The subtle amplitude of ME-related muscle motions makes them difficult to observe with the naked eye. In this study, we used motion magnification technology to make the ME sequence more clearly visible [36]. Wu et al. [37] proposed a method called Eulerian Video Magnification (EVM). The first step of this algorithm is spatial filtering, where the video sequence is decomposed into a multi-resolution pyramid. In the second step, time domain bandpass filtering is applied to each scale image to obtain several frequency bands of interest. In the third step, the filtering results are amplified. The signal of each frequency band is approximated by Taylor series, then the spatial frequency band as-filtered is given an amplification factor α to amplifying the interested signal linearly. Finally, a composite image is constructed and an enlarged image is synthesized.

The information in ME sequences mainly includes movements of the eyebrows, eyes, nose, and mouth. These organs move slightly with changes in mood while most areas of the face (the “background”) remain unchanged. To determine the emotional state, we need to magnify the small movements of these organs/muscles. Most of the subtle motions are concentrated in the low-frequency region, so we selected a bandwidth filter with frequency of 0.1–0.4 HZ in this paper. The filter has relatively narrow frequency band which allowed us to effectively amplify the motion information while avoiding amplifying any high-frequency noise information.

**Figure 6:**
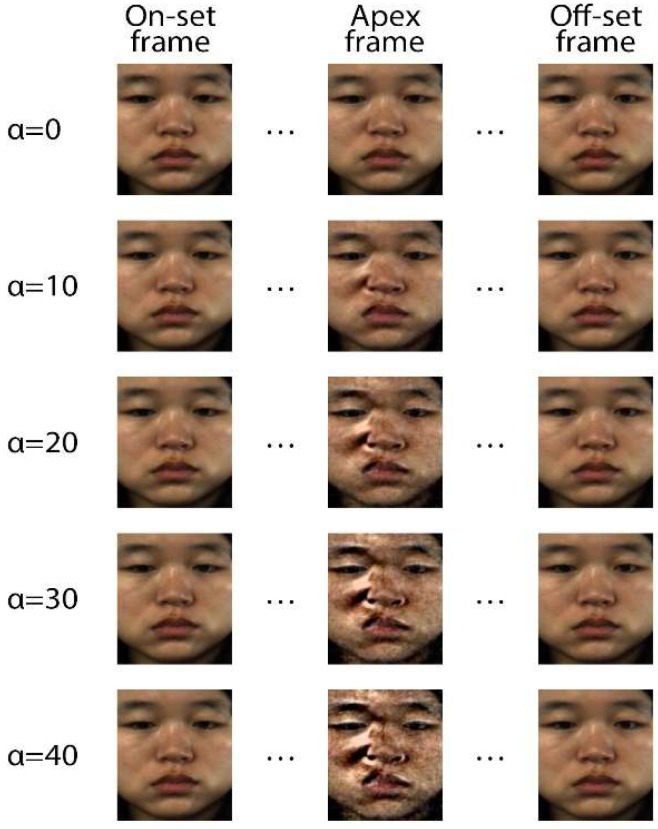
Comparison of ME sequences at different magnification factors.

Furthermore, we compare the movement results of the same emotion “adjust” according to different magnification factor α, as shown in Figure 6. The results show that when α=10, we could not clearly observe the facial movement information as the MEs were still too subtle, and the magnification coefficient was not sufficient to reflect motion in the face. When α=20, the overall effect was ideal and there was little noise interference. When α=30, the effect of noise was clearly observed but it still within an acceptable range. At magnification factor of 40, noise severely affected the visual effect and the facial image was distorted. The EVM method can be used to effectively avoid noise while enlarging the facial motions though distortion does occur once the magnification reaches a certain threshold.

There are a large number of small signals in the ME sequences. These small movements are likely to be ignored if they cannot be detected. Using the EVM algorithm, we were able to magnify the small motion in the video without estimating the motion in advance. Compared to other motion magnification algorithms, our algorithm has lower computational complexity and works in less computing time because it does not require motion estimation.

#### 2.2.2. Apex Frame Extraction

Ekman indicated that snapshot taken at a point when the expression is at its apex can easily convey the emotional message [9]. In other words, the apex frame provides the most useful information for facial expression recognition. Because ME sequences are taken by high-speed cameras, a large number of redundant frames are generated and some invalid emotional information is contained within these frames even after applying EVM technology [38]. If we input these ME sequences into the deep network, a large number of redundant and invalid features will be generated. Subsequently, these features will greatly reduce the accuracy of the network and increase the time cost of the operation. Extracting useful frames and discarding redundant frames eliminates useless information while minimizing computational cost and enhancing overall operational efficiency.

Liong et al. [18] extracted features from the onset frame, apex frame, and offset frame of a video for classification and recognition, and these three frames can provide enough information for recognition. In this study, we located the apex frames in ME sequences according to Li et al.’s [19] method based on the frequency domain, which functions differently from other apex frame extraction algorithms [4,18,39]. It is difficult to detect MEs in the spatial domain based on their inherent characteristics. Li et al. analyzed the signal changes of MEs in frequency domain and used 3D Fast Fourier Transform (3D-FFT) to transform sequences into signal representations in the frequency domain, then further located the apex frame in the video according to the maximum frequency amplitude. This technique encompasses necessary dynamic information and can be operated faster than spatial-domain-based techniques.

In this paper, we extracted apex frames from the ME sequences after magnification treatment and combined relevant AUs in FACS to generate the CEMD. Each emotion category in the CEMD contains three images, i.e., the onset frame, apex frame, and offset frame.

### 2.3. Extraction of Optical Flow Features

In this section, we used the Total Variation-L1 (TV-L1) optical flow algorithm [40] to calculate the motion information between the onset frame and the apex frame. Then, the corresponding optical flow feature map was obtained or subsequent deep network learning accordingly. The algorithm preserves the discontinuity of the image as well as the edge features of the image and other feature information. The optical flow uses the L1 norm to enhance the robustness of registration.

The optical flow feature reflects the instantaneous velocity of the corresponding points on the imaging plane. If it is used to estimate the point displacement caused by the disparity of gray levels between the continuous frames in ME sequences, it can accurately reflect changes in facial movements [41]. Optical flows are used to estimate the relative motion between two frames of time t and t + ∆t. The optical flow method needs to satisfy the following constraints:

(1) The brightness value of the pixels in the picture remains unchanged in a very short time;
(2) The motion scale of the pixel block in the picture is very small compared with the time variation between the continuous frames;
(3) The pixels in the neighborhood of an image have the same motion.

Assuming that there are two consecutive frames $I_{0}$ and $I_{1}$, X=(x,y) is a pixel point of frame $I_{0}$. The energy function of TV-L1 optical flow model is as follows:

(1) $$E=\int_{\Omega }\left\{\lambda \left|\mu \nabla I_{1}+I_{1}\left(X+U_{0}\right)-U_{0}\nabla I_{1}-I_{0}\right|+\left|\nabla U\right|\right\}dx$$

where U = (u, v) is a 2D optical flow field; ∇u and ∇v are the second derivatives of u and v. The parameter λ is the weight constant of the data item. The first item of Equation (1) is the data constraint item, which represents the gray value difference between the two frames of a single pixel point. The second is the constraints of motion regularization, which assumes that the motion is continuous.

To calculate the TV-L1 optical flow, we used a two-way numerical analysis mechanism based on image denoising to minimize the total variational optical flow energy function. The pixels near $X+U_{0}$ in the image $I_{1}$ were linearized:

(2) $$I_{1}\left(X+U\right)=I_{1}\left(X+U_{0}\right)+\left(U-U_{0}\right)\nabla I_{1}\left(X+U_{0}\right)$$

The first order Taylor expansion approximation of $I_{1}\left(X+U\right)$ can be plugged in next to minimize the calculation burden while seeking an exact solution. We used the two-way solution method to compensate for the error caused by the linear approximation by alternating updating variables. Equation (2) is introduced into Equation (1) as follows:

(3) $$E=\int_{\Omega }\left\{\lambda \left|U\nabla I_{1}+I_{1}\left(X+U_{0}\right)-U_{0}\nabla I_{1}-I_{0}\right|+\left|\nabla U\right|\right\}dx$$

Ordering $\rho U=I_{1}\left(X+U_{0}\right)+\left(U-U_{0}\right)\nabla I_{1}-I_{0}$ and introducing an additional variable *U*′ transforms Equation (3) into the following minimizing convex function:

(4) $$E=\int_{\Omega }\left\{\lambda \left|\nabla U\right|+\frac{1}{2\theta }{\left(U-U^{\prime }\right)}^{2}+\rho U\right\}dx$$

where *θ* is a small constant and $U^{\prime }$ infinite approximation to U. Equation (4) can be optimized by alternating updates of $U^{\prime }$ and U in the iteration. Finally, the solution of Equation (4) can be obtained by the following threshold equation:

(5) U′=U+{λθI1X,   if ρ(U)<− λθ(I1X)2−λθI1X,    if ρ(U)>− λθ(I1X)2−ρUI1X,  if |ρ(U)|≤λθ(I1X)2

**Figure 7:**
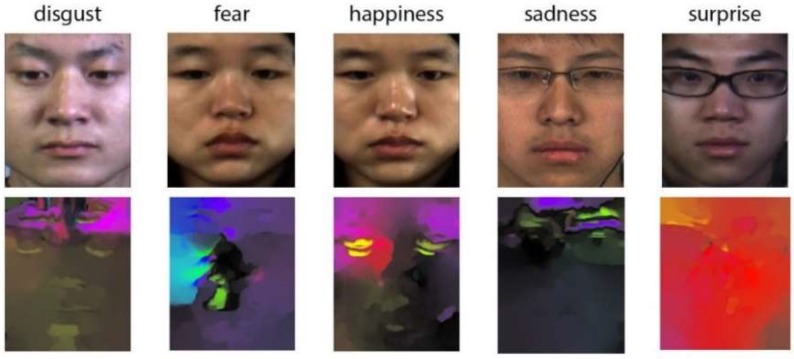
Optical flow maps of six MEs in CASME Ⅱ database.

Compared to other optical flow models [5,6], the TV-L1 model has several advantages in calculating the optical flow features of MEs. The total variational regularization term preserves the discontinuity of the displacement field and protects the edge information from blurring in the diffusion process. The data item also uses robust L1 norm ensure robustness to brightness changes. As shown in Figure 7, we calculated the optical flow features maps of several basic ME images. The optical flow feature accurately represents the movements of MEs.

### 2.4. Compound ME Recognition Based on CNN

Compared to traditional ME feature technologies based on manual extraction [3,4,5,6], deep networks have more powerful data fitting ability and more adaptable to the motion characteristics of ME sequences. Traditional expression recognition methods based on deep learning serve to input ME images or related optical flow feature maps directly into the network [19,20,21]. It is difficult for the network to distinguish the emotional categories of MEs because of their subtlety and similar muscle contraction units. In this study, we input the optical flow feature map of the ME sequences disposed by EVM into a CNN—this allowed us to secure a network that effectively judges ME motion characteristics and makes the extracted higher-level features more discriminative. The scope of MEs was also extended from six basic emotional categories to 18 compound emotional categories. This method accurately describes the emotional states encountered by individuals, and makes ME recognition more practical on the whole.

**Figure 8:**
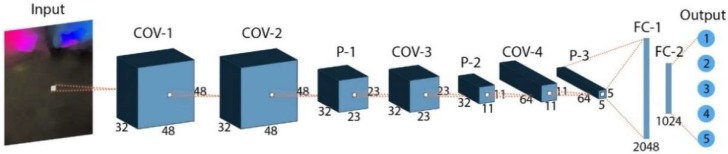
Overall framework of proposed network.

The network structure proposed in this study is shown in Figure 8. Here, a 1 × 1 convolution layer was added after the input layer, which increases the non-linear expression of input data, deepens the network to a certain extent, and also improves the expressive ability of the model. This operation does not increase the computational load of the model.

The higher-level features extracted from CNN were transferred to the output layer through the Full Connection (FC) layer. The number of neurons in the output layer is the same as the number of classes. In this study, there were three (basic ME) and seven (compound ME) neurons in the output layer. The FC layer maps network features to the label space of the samples and makes a prediction. We used the loss function to measure the error between the predicted value and the real sample:

(6) $$L_{softmax\;loss}=-\frac{1}{N}\overset{N}{\underset{i=1}{\sum }}\log\left(\frac{e^{h_{y_{i}}}}{\sum_{j=1}^{C}e^{h_{j}}}\right)$$

where N denotes the number of classified samples, $y_{i}$ is a real label of input sample $x_{i}$, $h_{j}$ is the output of the network (the predicted results of samples), and C is the number of classes. The loss function transforms the output into probability form via exponential transformation. Because the volume of the ME database is very small, we used dropout technology to alleviate the complex synergistic adaptation effect and reduce the dependence between neurons. This technology also minimizes the likelihood of network over-fitting.

## 3. Results and Discussion

### 3.1. Basic ME Databases

The development of automatic ME recognition systems depends largely on the spontaneous and representative databases. In this study, we investigated five representative databases (CASME I, CASME II, CAS(ME)^2^, SMIC, SAMM) (Table 3) in terms of frame rate, emotional categories, and resolution. We also tested the CMED on these databases. In addition, we also introduced the CMED database produced by this paper in detail.

### 3.2. CMED

According to the spontaneous ME databases described above, we generated a novel compound ME database in this paper. The CMED is composed of four spontaneous MEs databases: CASME I, CASME II, CAS(ME)^2^, and SAMM (the SMIC database lacks the corresponding AU coding, so we could not use it to generate composite expressions).

Firstly, we standardized images by detecting, aligning, and tailoring [42] and finally adjusting their resolution to 150 × 190. Then, the EVM algorithm is applied to magnify the muscle movements of these ME images for AU-analysis and synthesis of composite MEs. However, the sample sizes and emotional distribution in these databases are very uneven, which makes it difficult for recognition. For example. in CASME II, 64 samples express the emotion of “disgust” while only 32 samples express “happiness”. In order to solve this problem, it is necessary to divided the traditional ME databases into new classes according to the emotional states. In basic MEs part, we use three emotional classes for recognition: Negative (Neg), Positive (Pos), and Surprise (Sur). As shown in Table 4, we divide compound emotions into four categories: Positively Surprised (PS), Negatively Surprised (NS), Positively Negative (PN), and Negatively Negative (NN). In summary, seven emotional classes in CMED are supplied for recognition and data analysis.

In this paper, we will experiment on two databases: the basic ME database (including CASME I, CASME II, CAS(ME)^2^, SMIC, SAMM) and CMED (generating by CASME I, CASME II, CAS(ME)^2^, SAMM). The former has three classes (Neg, Pos, Sur), and the later has seven classes (Neg, Pos, Sur, PS, NS, PN, NN).

### 3.3. Experiment Settings

**Figure 9:**
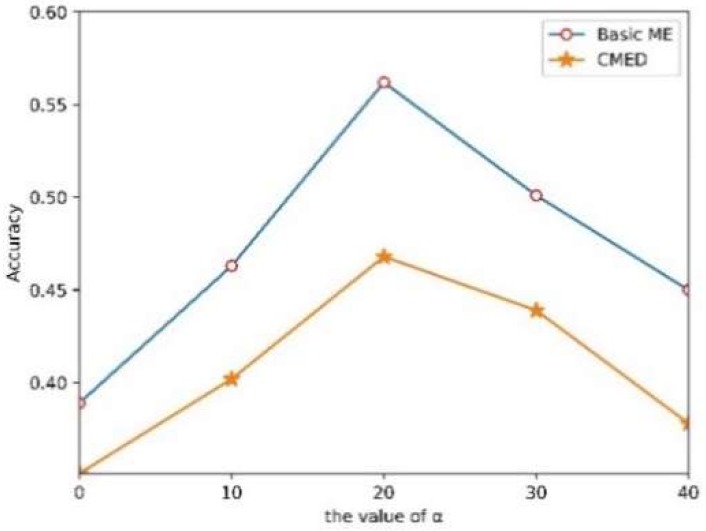
Recognition performance using different magnification factor.

Firstly, we adjust the resolution of basic and compound ME images to 150 × 190 according to the above experiments. Secondly, we set the parameters of the EVM method for enlarging the subtle motions. Both Laplacian pyramid and Gauss pyramid can be used for spatial filtering. The resulted show that this article needed to enlarge the muscle movements corresponding to MEs, so we chose the Laplace pyramid to construct the baseband of different spatial frequencies. Similarly, time-domain filtering can select different bandpass filters according to different requirements. Therefore, we chose the Butterworth band-pass filter because the time-frequency analysis of the amplification results is not necessary after the amplification motion. The motion range of MEs is very subtle, so too small magnification factor does not reflect the contraction of muscles. However, if α is too large, the image distortion and noise caused by the camera may lead to the decline of experimental accuracy. As shown in Figure 9, we compared the effects of different amplification coefficients on the experimental accuracy in basic ME database and CMED. In this experiment, we only input the enlarged images of MEs into CNN, and the results show that α=20 is the appropriate magnification.

**Figure 10:**
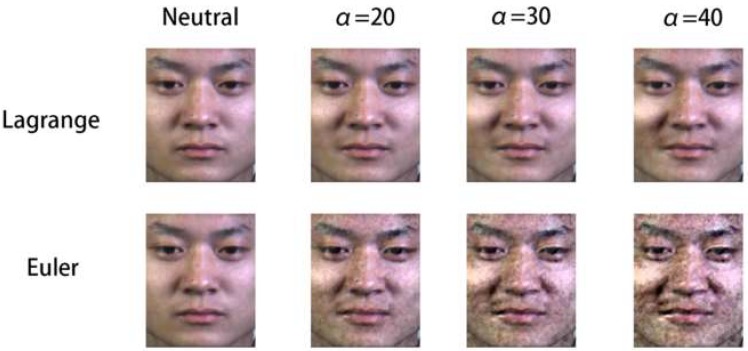
Comparison of different magnification method.

In order to prove the validity and discriminability of EVM algorithm, the video magnification technologies based on Lagrange perspective and Euler perspective was compared in Figure 10. The result shows that the effect of Eulerian amplification is more obvious. The muscle movement of micro-expression is very subtle and hard to be detected by the naked eye. However, the basis of synthesizing compound micro-expression is to show the facial muscle contraction of each emotion. This paper uses the EVM to judge the emotion category and action units (AUs). Although Lagrange technology can reduce the influence of noise and blur, its amplification effect on micro-motion is poor, which deviates from the original intention of compound micro-expression synthesis. Furthermore, the corresponding filter can also avoid the blur and noise amplification. In this paper, the main requirement of video amplification technology is to enlarge the subtle changes of micro-expression sequences, so we choose EVM algorithm.

Next, we used the TV-L1 algorithm to extract optical flow features from the onset frame and the apex frame of enlarged ME images. The parameters of the proposed algorithm were taken from in the literature [41]: τ=0.25, λ=0.05, θ=0.3, ε=0.01, η=0.5, $N_{\mathrm{scales}}\;=3$, and $N_{\mathrm{warpings}}\;=5$. Among them, the parameters λ and $N_{\mathrm{scales}\;}$ are the two most influential. λ is the weight that affects the regular term and data term. The regular term of the optical flow model hinders changes in the optical flow field to ensure a smooth optical flow field, so if the value λ is larger, the influence of the optical flow constraint term is greater. As long as there is a little deviation from the optical flow constraint, this will lead to functional divergence. On the contrary, a smaller λ results in less influence on the flow constraints. $N_{\mathrm{scales}}$ was used to create the pyramid of images. A small value detects only pixel-level movement and a large value detects larger displacement.

**Figure 11:**
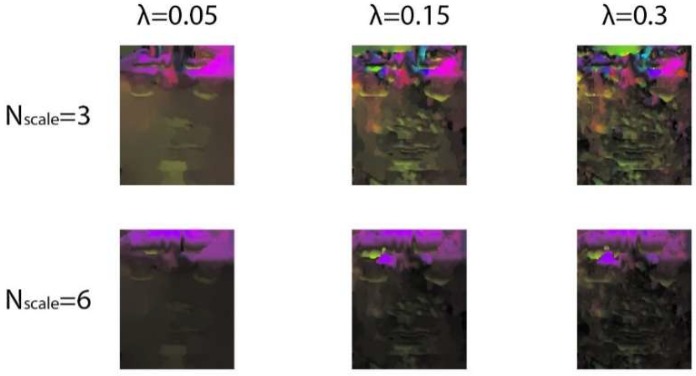
Optical flow feature maps with different λ and $N_{\mathrm{scales}}$.

Figure 11 shows the optical flow feature maps corresponding to different λ and $N_{\mathrm{scales}}$ of the emotion “disgust” in the CASME II database. When λ = 0.3, the influence of optical flow constraints is large. The TV-L1 optical flow algorithm is very sensitive to noise, which leads to optical flow graph instability. The small displacement and noise produced by the camera makes these unnecessary movements particularly obvious under this condition. The motion amplitude of MEs is so slight that $N_{\mathrm{scales}}\;=6$ cannot detect the muscle contractions, resulting in inaccurate motion representation on the optical flow map.

In the CNN network, we have implemented our model on Anaconda3 with Python3.6 and Tensorflow at the backend. The model was trained and tested on CPU with CORE i5 7th Gen and a GPU Server with GTX 950m (2GB). Before training the network, we need to process the optical flow feature maps. Firstly, we need to change the resolution of these data to 48 × 48. Then, we randomly mess up the data and adopt the mini-batch training mechanism to improve the convergence velocity and the efficiency of test set. Generally speaking, the larger the batch size is, the more accurate the descending direction is, and the smaller the training vibration is. However, a large batch size value is easy to make the network fall into a local optimum. Hence, both the networks are trained for 1500 epochs with batch size of 10. Finally, we divide the data set into training set, verification set and test set, and transform them into TFRecord format to save memory space. The parameter settings for each layer of the network are shown in Table 5.

As shown in the Table 5, a convolution layer of 1 × 1 was added after the input layer to increase the non-linear expression of the input and improve the expressive ability of the model. In this paper, small convolution kernels such as 5 × 5 and 3 × 3 are used to enhance the network capacity and model complexity. The zeros padding operation can make full use of the edge information and avoid the sharp reduction of input size. In order to avoid losing the input responses, the pooling layer is generally set to a smaller value. When setting the learning rate, the initial value should not be too large. In this paper, the value is set to 0.01. And the learning rate slows down with the increase of epochs, so it decreases 10 times smaller after every 10 epochs. At the same time, the Batch Normalization (BN) is used to regulate the input layers and fix the mean and variance of each layer. This measure not only speed up the convergence, but also solve the problem of “gradient dispersion”. To minimize over-fitting, we added a dropout operation after two FC layers and set the ratio to 70%. In the softmax-classification layer, we set up two classifications: basic MEs and compound MEs. In the basic ME recognition stage, we split the output into three classes. In the CMED stage, we split the output into seven classes. We used a left-one cross-validation method to test the robustness of the proposed algorithm. Ultimately, Leave-One-Subject-Out (LOSO) cross-validation is used in our evaluation to prevent subjective errors during learning.

### 3.4. Analysis and Discussion

In this section, we analyzed the performance of the proposed algorithm including comparison against other state-of-the-art algorithms, and also compared the accuracy of different epoch values, as shown in Table 6. At same time, F1-Measure was used to evaluate the performance of the proposed model. The result reflects that accuracy rate and F1-Measure value increased with an increasing number of rounds, but when epoch value = 1000, both indicators decreased. This is because the amount of data in basic/compound ME databases is very small. The number of weight-update iterations in the neural network increased as the epoch number increased, while the model entered an over-fit state. The epoch size is related to the diversity of the dataset. The number of emotional categories and samples in the two ME databases discussed in this paper are both small, so we were careful not to over-train the network.

**Figure 12:**
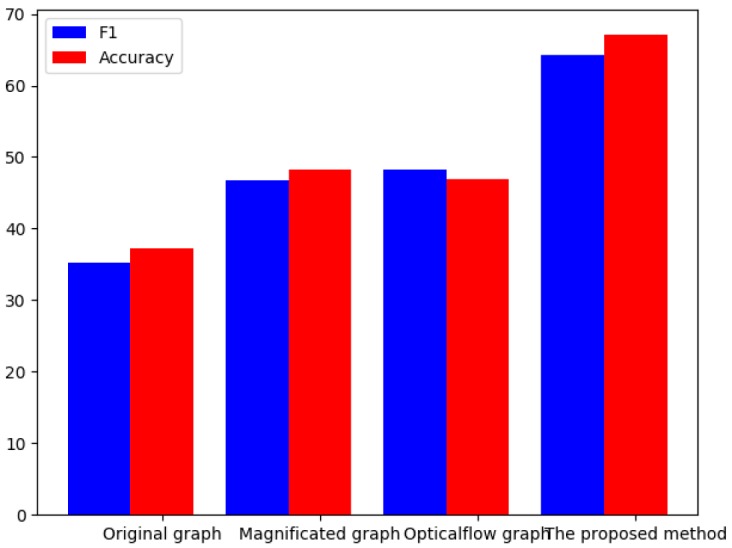
Recognition performance using different input graph on CMED. Magnified not magnificated.

In order to prove the effectiveness of the proposed algorithm, we input four different feature images (original graph, magnified graph, optical-flow graph and the proposed method) into CNN and get the histogram as shown in Figure 12. The experiment indicates that this algorithm not only enlarges the subtle muscle movements of MEs on the image-display level, but also extracts the optical flow features, which provides a more accurate and robust image basis for subsequent depth feature extraction and recognition.

As shown in Table 7, the proposed algorithm was tested on CASME II, SMIC, and SAMM databases and compared with other the state-of-the-art algorithms. The tested algorithms can be roughly divided into two categories: manual and deep learning feature extraction methods. The deep learning algorithms outperform the manual extraction algorithms on all three databases. However, their accuracy degrades as the deep network layer quantity increases. This is due to the inherent attributes of ME databases.

The sample sizes and sample classes of the databases we used are very small. Their sample distribution is also very uneven, which prevents the deep network from executing feature extraction as effectively as possible and readily leads to over-fitting. The proposed algorithm has some notable advantages in computational performance. Firstly, we did not directly use ME images to extract deep features but rather input the optical flow feature map generated by the enlarged onset and apex frames into the network, which avoids redundancy. The optical flow graph strengthens muscle contraction recognition during ME movement and is more discriminative. We also used a shallow neural network to extract and recognize optical flow map features, which not only extracts higher-level features from the deep network effectively but also alleviates over-fitting.

Table 8 summarizes the properties of all the neural networks mentioned in Table 7, including depth, image input size and execution time. The execution time is the testing time of a single subject. This value is related to the depth and complexity of a network. The network proposed in this paper is better than OFF-ApexNet and STSTNet in both depth and model complexity. As a result, our network spends more time on testing a sample. Furthermore, the network designed in this paper combines the characteristics of micro-expression so that it is better than the traditional depth networks in execution time and accuracy.

**Figure 13:**
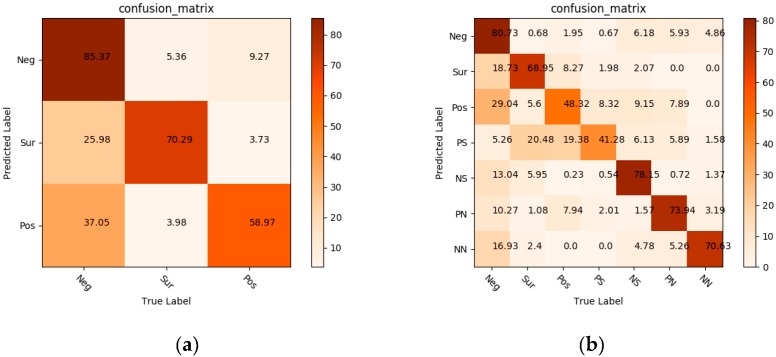
The measurement of confusion matrix: (**a**) the basic ME database; (**b**) the CMED.

We calculated the confusion matrices on two databases, the basic ME database and CMED. As shown in Figure 13, the recognition accuracy of negative emotions is higher than that of the other two emotional categories on the basic ME database, which is due to the largest number of negative emotions in the mixed datasets (Negative samples = 329, Positive samples = 134, Surprise samples = 113). On the other hand, there is a certain degree of misjudgment because surprise generally triggers the initial state of all other emotions.

Figure 13b shows the accuracy of various emotional categories in the CMED. The emotional categories with larger sample sizes were recognized higher recognition rates. The algorithm was particularly accurate on “negative” and “negatively surprised” classes, because of the large number of samples compared with other categories. Compound emotions depend on the AUs of basic emotions, so there is a subordinate relationship between them to a certain extent. It is difficult for machines to distinguish compound MEs and their subsets. For example, “negative” is the dominant component of “negatively surprised”, so these two kinds of emotional states share information and AUs among themselves. The experimental results have indicated that PN had a 10.27% misunderstanding rate on negative emotions and a 7.94% misunderstanding rate on surprise emotions. This is because this compound MEs are composed of these two basic emotions and share the facial movement information. The compound MEs were misrecognized at a higher rate on their main basic MEs, but not on unwanted emotional states. From the Figure 12, we can also understand that there are some differences between compound MEs and basic MEs. The generation and recognition of CMED broaden human understanding and recognition of emotion. Overall, the proposed method executed satisfactory recognition performance on the basic ME databases and CMED.

In the experimental part, this paper adopts the method of training in one database and testing in the same database. Although good recognition results have been achieved, the experiment does not test the effect of cross dataset. Therefore, the subsequent experiments will focus on the way of training on one dataset and testing on another one, but the expected experimental results of this method may not be satisfactory. Although the deep network technology improves the low accuracy rate of micro-expression recognition, the existing rate is not enough to support the future application requirements. We think there may be two reasons. Firstly, the low accuracy of this paper is due to the lack of enough data to adjust the parameters of the deep model. Obviously, the training and debugging of deep learning network needs a large number of samples, and the sample-number of our databases is too small. Although we can use some methods to increase the number of samples, due to the characteristics of micro-expression, it is difficult for non-professional person to label the emotions, so the new samples are often unsatisfactory. The second drawback of this paper is that only using the optical flow feature map to extract the deep features does not conform to the characteristics of micro-expressions. We need to adjust the input of the network according to the features of the data source. We can use multi-level input mode to train the depth network and refine each level network to meet the needs of micro expression recognition.

## 4. Conclusions

The experimental results and analysis presented above suggest that compound MEs and their corresponding components (i.e., basic MEs) have a high degree of connectivity and unity. Each compound expression has its corresponding dominant emotional category (for instance, “positive” is the dominant category in the emotion of “positively surprised”) and each basic ME is a constituent element of compound emotion. We performed image processing on basic/compound MEs in this study. EVM technology can magnify subtle muscle contractions, while the optical flow feature well represents the emotional production process. We used a shallow network to extract and classify the features of the optical flow to secure a series of experimental results. We expanded the emotional categories of MEs and used computer vision and deep learning algorithms to test the proposed method as well.

In this study, the mainstream databases of spontaneous MEs were investigated and a corresponding compound ME database was generated. However, this database was not created by inducing emotions by videos in laboratory settings, then recruiting professional psychological researchers to label the emotional categories. In the future, we hope to generate a database of spontaneous MEs based on complex emotions for further analysis. In the future, we will improve the algorithm to adapt to the application of micro-expression recognition. First of all, micro-expression is mainly video data. The follow-up work will focus on extracting temporal and spatial information of micro-expressions using deep network. We will combine the long-term memory network and convolution network to retain the characteristics of the micro-expression sequences and verify them on the database. Next, according to the research of psychologists, micro-expression has the necessary morphological regions corresponding to emotion. These regions are more discriminative in micro-expression recognition, so we aim to design a deep selection network to screen out the necessary morphological regions with discriminative ability and use these regions to recognize basic micro-expression and compound micro-expression.

## Figures and Tables

**Table 1:** Prototypical AUs of 18 emotions (6 basic emotions and 12 compound emotions) described by Martinez [10].

Emotion	Prototypical AUs
Happiness	12, 25, 6
Sadness	4, 15 [1 (60%), 6 (50%), 11 (26%), 17 (67%)]
Fear	1, 4, 20, 25 [2 (57%), 5 (63%), 26 (33%)]
Angry	4, 7, 24 [10 (26%), 17 (52%), 23 (29%)]
Surprise	1, 2, 25, 26 [5 (66%)]
Disgust	9, 10, 17 [4 (31%), 24 (26%)]
Happily surprised	1, 2, 12, 25 [5 (64%), 26 (67%)]
Happily disgusted	10, 12, 25 [4 (32%), 6 (61%), 9 (59%)]
Sadly fearful	1, 4, 20, 25 [2 (46%), 5 (24%), 6 (34%), 15 (30%)]
Sadly angry	4, 15 [6 (26%), 7 (48%), 11 (20%), 17 (50%)]
Sadly surprised	1, 4, 25, 26 [2 (27%), 6 (31%)]
Sadly disgusted	4, 10, 25 [1 (49%), 6 (61%), 9 (20%), 11 (35%), 15 (54%), 17 (47%)]
Fearfully angry	4, 20, 25 [5 (40%), 7 (39%), 10 (30%)]
Fearfully surprised	1, 2, 5, 20, 25 [4 (47%), 26 (51%)]
Fearfully disgusted	1, 4, 10, 20, 25 [2 (64%), 5 (50%), 9 (28%), 15 (33%)]
Angrily surprised	4, 25, 26 [5 (35%), 7 (50%), 10 (34%)]
Angrily disgusted	4, 10, 17 [7 (60%), 9 (57%), 24 (36%)]
Disgustedly surprised	1, 2, 5, 10 [4 (45%), 9 (37%), 17 (66%), 24 (33%)]

**Table 2:** Prototypical AUs of 6 basic MEs.

Emotion	Prototypical AUs
Happiness	6, 12
Sadness	1, 4, 15
Fear	1, 4, 20
Anger	4, 7, 43
Disgust	4, 7, 9, 25, 26
Surprise	1, 2, 5

**Table 3:** Basic information of databases used in experiment.

		CASME I	CASME II	CAS(ME)^2^	SMIC-HS	SAMM
**Year**		2013	2014	2016	2013	2018
Participants		19	24	22	16	28
Frame rate (fps)		60	200	30	100	200
FACS coded		Yes	Yes	Yes	No	Yes
Face resolution		150 × 190	280 × 340	190 × 230	130 × 160	960 × 650
Emotion classes		7	5	4	3	7
Expression	Negative	52	88	28	70	91
	Positive	9	32	16	51	26
	Surprise	20	25	10	43	15
	Total	81	145	54	164	132
Ground-truth (index)	Onset	Yes	Yes	Yes	Yes	Yes
	Apex	Yes	Yes	Yes	No	Yes
	Offset	Yes	Yes	Yes	Yes	Yes

**Table 4:** Compound ME database.

		CASME I	CASME II	CAS(ME)^2^	SAMM	CMED
Pos	Happiness	9	32	15	26	82
Neg	Disgust	44	64	16	9	233
	Fear	2	2	4	8	
	Anger	-	-	7	57	
	Sadness	6	7	1	6	
Sur	Surprise	20	25	10	15	70
PS	Happily surprised	16	18	20	20	74
NS	Sadly surprised	5	19	-	7	236
	Fearfully surprised	-	-	8	8	
	Angrily surprised	-	-	12	26	
	Disgustedly surprised	62	73	16	-	
PN	Happily disgusted	6	143	13	35	197
NN	Sadly fearful	2	-	-	7	158
	Sadly angry	-	-	-	18	
	Sadly disgusted	28	52	-	1	
	Fearfully angry	-	-	2	2	
	Fearfully disgusted	-	-	5	-	
	Angrily disgusted	-	-	10	31	

**Table 5:** Network structure.

Layer	Filter Size	Stride	Output Size	Dropout
Input			48×48×3	
Conv-1	1×1	1	48×48×12	
Conv-2	5×5	1	48×48×12	
Pool-1	3×3	2	23×23×12	
Conv-3	3×3	1	23×23×12	
Pool-2	3×3	2	11×11×12	
Conv-3	5×5	1	11×11×24	
Pool-3	3×3	2	5×5×24	
FC-1			1024×1	70%
FC-2			1024×1	70%
Output				3×1
				7×1

**Table 6:** Recognition accuracy and F1-measure evaluated on basic/compound ME databases.

Epoch	Basic ME		CMED	
	Accuracy (%)	F1-Measure	Accuracy (%)	F1-Measure
100	76.19	0.7304	66.06	0.6353
300	78.52	0.7518	66.93	0.6384
500	80.64	0.7724	67.15	0.6418
1000	77.03	0.7401	65.14	0.6267

**Table 7:** Recognition performance: Accuracy (%) on the CASME II, SMIC, and SAMM databases for state-of-the-art methods and the proposed method.

Method	CASME II (5 Classes)	SMIC (3 Classes)	SAMM (7 Classes)
LBP-TOP [3]	39.68	43.73	35.56
LBP-SIP [17]	43.32	54.88	-
STLBP-IP [22]	59.51	57.93	-
STCLQP [23]	58.39	64.02	-
OSF [6]	-	31.98	-
OSW [25]	41.7	53.05	-
MDMO [5]	44.25	-	-
Bi-WOOF [39]	57.89	61.59	51.39
AlexNet [43]	83.12	63.73	66.42
GoogLeNet [44]	64.14	55.11	59.92
VGG 16 [19]	82.02	59.64	47.93
OFF-ApexNet [20]	86.81	66.95	53.92
STSTNet [21]	86.86	70.13	68.1
Proposed method	87.01	69.79	70.18

**Table 8:** Properties of the neural networks.

Network	Depth	Image Input Size	Execution Time (s)
AlexNet [43]	8	227×227×3	12.9007
GooLeNet [44]	22	224×224×3	29.3002
VGG 16 [19]	16	224×224×3	95.4436
OFF-ApexNet [20]	5	28×28×2	5.5632
STSTNet [21]	2	28×28×3	5.7366
The Proposed Method	11	48×48×3	10.9402
